# Primary versus Metastatic Gastrointestinal Melanoma: A Rare Case and Review of Current Literature

**DOI:** 10.1155/2016/2306180

**Published:** 2016-08-29

**Authors:** Malorie Simons, Jason Ferreira, Rashna Meunier, Steven Moss

**Affiliations:** ^1^Department of Medicine, Rhode Island Hospital and Warren Alpert School of Medicine of Brown University, Providence, RI, USA; ^2^Dartmouth-Hitchcock Gastroenterology and Hepatology, Lebanon, NH, USA; ^3^Division of Surgical Pathology/Gastrointestinal and Liver Pathology, University of Massachusetts, Boston, MA, USA; ^4^Division of Gastroenterology, Rhode Island Hospital and Warren Alpert Medical School of Brown University, Providence, RI, USA

## Abstract

Gastrointestinal (GI) melanomas are a rare diagnostic entity. Although there have been cases of melanomas solely in the GI tract, many debate their true origin: the gut versus a distant, undetected primary lesion that regressed known as melanoma of unknown primary. We present a case that involved diagnosing a GI melanoma and then backtracking to find a possible primary source. We review the most recent literature regarding possible etiologies of primary GI melanomas and how to differentiate whether it has a primary, metastatic, or unknown origin.

## 1. Introduction

Gastrointestinal (GI) melanomas are unusual, and if found, require thorough investigation. Most GI melanomas are metastatic from an oculocutaneous primary lesion but, although rare, primary GI melanomas have been found in all levels of the GI tract [1]. Some believe that GI melanomas manifest from melanoma at another site, and if not found, are called melanoma of unknown primary (MUP). In a large retrospective review of patients seen at Massachusetts General Hospital and the Dana-Farber Cancer Institute, only 2.5% had MUP [2].

We present a patient with GI melanoma, first found in the colon and then a suspicious inguinal lymph node chain giving a clue to a possible site of origin; however, by this time, the disease was widely metastatic and therapeutic options were limited. This case emphasizes that melanoma found in the GI tract is a challenging diagnostic entity that requires thorough diagnostic investigation.

## 2. Case Report

**Figure 1:**
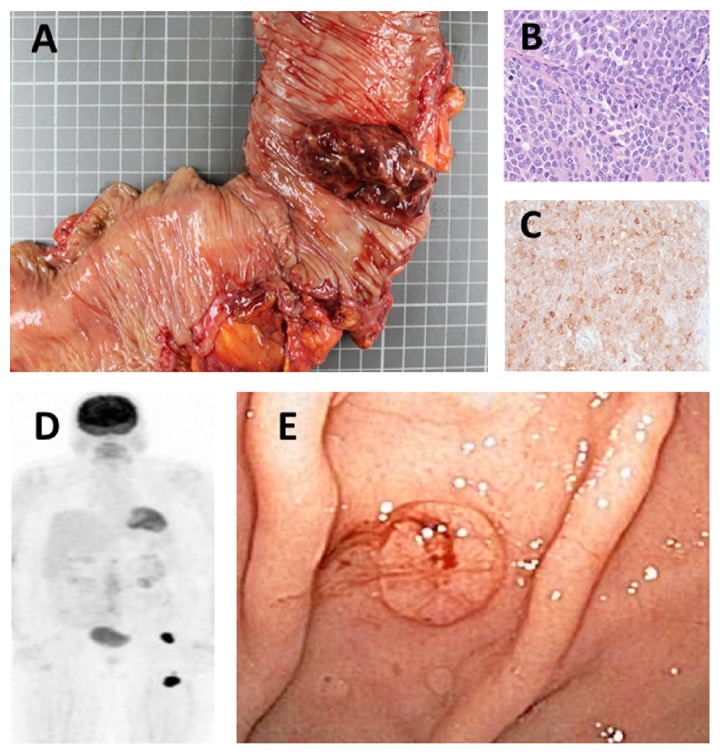
Extended right hemicolectomy specimen containing a 5.6 cm exophytic, partially obstructing lesion (a). Histology of colon lesion showing sheets of medium- to large-sized tumor cells with irregular nuclear contours and vesicular nuclei, intermixed with numerous mitotic figures ((b), hematoxylin-eosin, 400x). Immunohistochemistry of colon lesion diffusely positive for melan-A (c). PET scan showing areas of metastatic disease (left renal mass, L1 vertebral body), but prominent intensity of the left inguinal region and left anterior thigh (d). Endoscopy showing one of many pigmented lesions throughout the stomach (e).

An 82-year-old female with chronic obstructive pulmonary disease and diastolic cardiomyopathy presented with three weeks of fatigue, abdominal distention, and hematochezia. She was found to be anemic with bright red blood in her stool. Colonoscopy revealed a 5.6 cm partially obstructing, exophytic lesion near the hepatic flexure that was later surgically resected with an extended right hemicolectomy (Figure 1(a)). Pathologic exam revealed diffuse sheets of medium to large sized tumor cells with moderate nuclear pleomorphism, irregular nuclear contours, and vesicular chromatin (Figure 1(b)). By immunohistochemistry, the tumor cells were diffusely positive for melan-A (Figure 1(c)), confirming the diagnosis of malignant melanoma. Surgical specimen showed negative margins and no lymph node involvement, but positive lymphovascular invasion. Computed tomography (CT) scan of the chest, abdomen, and pelvis showed three small lung nodules and one kidney lesion for which metastatic disease could not be excluded. No abnormal adenopathy, including the inguinal region, was detected. Since no oculocutaneous primary could be identified via physical exam, a PET scan was performed and showed a suspicious area of lymph nodes in the left inguinal region and anterior thigh (Figure 1(d)). Multiple repeat skin exams failed to demonstrate a cutaneous primary lesion in that area. At that point, she was diagnosed with metastatic melanoma and refused aggressive treatments.

Six months later, she represented with severe symptomatic anemia (shortness of breath, fatigue) and melena. She requested a palliative red blood cell transfusion and possible intervention to stop any bleeding. Endoscopy showed multiple pigmented lesions in her stomach (Figure 1(e)) that were cauterized and biopsied. Pathology confirmed gastric melanoma. Given the extent of her disease, multiple comorbidities, and limitations to the treatments for widely metastatic GI melanoma, hospice care services were initiated and she expired 12 days later.

## 3. Discussion

Melanoma of the GI tract is a rare occurrence that can carry a poor prognosis. The primary site of the melanoma is usually the skin and metastases within the GI tract commonly occur in the liver, small intestine, colon, and stomach in decreasing order of incidence. In fact, 60% of those with melanoma will have GI tract metastases at the time of autopsy [3]. Of the noncutaneous melanomas, 20% arise from mucosal sites and of these, 25% are found in the GI tract [4].

The etiology of primary GI melanomas is unclear and speculative. One hypothesis suggests that the melanoma arises from the neural crest cells known to exist in the esophagus, stomach, small bowel, and anorectum.* In vitro*, these cells have a proclivity to develop into mature melanocytes, but this has yet to be established* in vivo*. Additionally, this idea precludes disease originating in the colon [4, 5]. A second, and more inclusive, hypothesis argues a defect in ectodermal differentiation and migration causing the melanocytes to reside inappropriately in the GI tract [6–8]. Despite these theories, the cause of primary GI melanomas remains a mystery.

Even with the possibility of melanoma arising from the GI tract, it is important to rule out an original site of metastatic disease. Approximately 2% of melanomas have an unclear primary source, including those that are present in the GI tract [9]. Many hypothesize that melanoma of the GI tract is a product of spontaneous regression of an unknown primary oculocutaneous lesion, also known as melanoma of unknown primary. In fact, in one study of 437 cutaneous melanomas, 12.3% showed at least partial regression [10]. Thus, a diagnosis of GI melanoma should encourage physicians to perform a thorough physical exam, including eyes, mouth, nasopharynx, genitalia, and anorectum. Major lymph nodes should also be examined to guide towards a possible primary site. If nothing is detected by physical exam, other tools should be used such as positron emission tomography (PET) scan.

This case report teaches us that melanoma first found in the GI tract is a challenging diagnostic entity that requires a thorough investigation to determine if it is a true primary lesion or metastatic from a distant site. We believe that our patient had metastatic melanoma from a regressed cutaneous lesion on her left thigh, given the suspicious unilateral inguinal lymphadenopathy and the lack of lymph node involvement after hemicolectomy that would suggest primary colon GI melanoma; however, this could not be proven. We recommend that after a GI melanoma is diagnosed, a physician must first perform detailed physical exam (including the nasopharynx, eyes, anus, and skin). If this does not uncover an oculocutaneous melanoma lesion, a PET scan is crucial to help discern if the GI melanoma is primary, metastatic, or of unknown origin.
